# Methylation of the Corticotropin Releasing Hormone Gene Promoter in BeWo Cells: Relationship to Gene Activity

**DOI:** 10.1155/2015/861302

**Published:** 2015-09-17

**Authors:** Xin Pan, Maria Bowman, Rodney J. Scott, John Fitter, Richard C. Nicholson, Roger Smith, Tamas Zakar

**Affiliations:** ^1^Mothers and Babies Research Centre, Hunter Medical Research Institute, New Lambton Heights, NSW 2305, Australia; ^2^Faculty of Health and Medicine, University of Newcastle, Callaghan, NSW 2308, Australia; ^3^Department of Obstetrics and Gynaecology, Second Affiliated Hospital, Chongqing Medical University, Chongqing, China; ^4^Molecular Medicine, Hunter Area Pathology Service, New Lambton Heights, NSW 2310, Australia; ^5^John Hunter Hospital, New Lambton Heights, NSW 2310, Australia

## Abstract

Corticotropin releasing hormone (CRH) production by the human placenta increases exponentially as pregnancy advances, and the rate of increase predicts gestational length. *CRH* gene expression is regulated by cAMP in trophoblasts through a cyclic AMP-response element (CRE), which changes its transcription factor binding properties upon methylation. Here we determined whether methylation of the *CRH* proximal promoter controls basal and cAMP-stimulated *CRH* expression in BeWo cells, a well-characterized trophoblastic cell line. We treated the cells with 8-Br-cAMP and the DNA methyltransferase inhibitor 5-aza-2′ deoxycytidine (5-AZA-dC) and determined the effects on *CRH* mRNA level and promoter methylation. Clonal bisulfite sequencing showed partial and allele independent methylation of CpGs in the *CRH* promoter. *CRH* mRNA expression and the methylation of a subset of CpGs (including CpG2 in the CRE) increased spontaneously during culture. 8-Br-cAMP stimulated *CRH* expression without affecting the increase in methylation. 5-AZA-dC decreased methylation and augmented 8-Br-cAMP-stimulated *CRH* expression, but it blocked the spontaneous increase of *CRH* mRNA level. We conclude that the *CRH* promoter is a dynamically and intermediately methylated genomic region in BeWo cells. Promoter methylation did not inhibit *CRH* gene expression under the conditions employed; rather it determined the contribution of alternative cAMP-independent pathways and cAMP-independent mechanisms to *CRH* expression control.

## 1. Introduction

CRH is a 41-amino-acid peptide hormone synthesized in the paraventricular nucleus of the hypothalamus. Its function in the central nervous system is to stimulate the hypothalamic-pituitary-adrenal (HPA) axis as part of the stress response. CRH is also produced in peripheral tissues including the placenta of humans and hominine primates [1–3]. Placental CRH secretion results in an exponential rise of CRH concentration in maternal plasma during the third trimester of gestation. The rate of the increase is related to gestational length, since the rise of CRH level is accelerated in pregnancies ending with preterm birth, while the increase is retarded in pregnancies that continue after term. Because of this relationship, it is believed that the regulation of placental CRH production is linked to the mechanism that determines the length of pregnancy and triggers labour and delivery [4]. The mechanism regulating the exponential increase in placental CRH expression remains unclear although positive feedback by glucocorticoids and increasing numbers of syncytial cell nuclei are suggested explanations [5, 6].

The principal source of placental CRH is the trophoblast syncytium [7]. Spontaneous and agonist-induced syncytium formation by cytotrophoblasts is associated with CRH expression with cAMP being a strong stimulant of both syncytial differentiation and* CRH* gene activity [8]. Molecular studies using* CRH* promoter-reporter constructs indicated that transcription factor complexes bound to a consensus cyclic-AMP response element (CRE) at 224 bp upstream of the major transcription initiation site mediated the cAMP-stimulation, although a nonconsensus second promoter site was also implicated in the cyclic nucleotide response [8–11]. It has been suggested that these molecular interactions are involved in the gestational age dependent control of* CRH* gene activity [6].

Epigenetic chromatin modifications define cell-specific gene expression potential and alter gene expression patterns during cell differentiation and development [12]. Methylation of cytosines at CpG dinucleotides in DNA is a well-characterized epigenetic chromatin modification generally associated with closed chromatin structure and gene repression [13]. Furthermore, methylation of CpG sites within particular transcription factor binding sequences may modify transcription factor binding affinity and alter regulatory changes in gene expression [14–17]. The human* CRH* proximal promoter contains 9 CpG dinucleotides with one located within the methylation sensitive CRE sequence. In addition, the promoter is within 1000 bp distance from an intragenic CpG island, which corresponds to a “CpG island shore” region, the methylation of which is related to tissue specific gene expression [18]. Therefore, in the present investigation we have explored the possibility that methylation of the promoter contributes to the control of* CRH* expression in trophoblast cells. We used the BeWo choriocarcinoma cell line in the experiments, which is a well-characterized trophoblast model exhibiting dynamic DNA methylation as well as ability to syncytialise and increase* CRH* expression when stimulated with cAMP [19–21].

## 2. Materials and Methods

### 2.1. Cell Culture

The BeWo human choriocarcinoma-derived cell line was obtained from the American Type Culture Collection (Manassas, VA, USA) (ATCC# CCL-98). Cells passaged fewer than 20 times were used in the experiments. The culture medium was DMEM/F12 (with HEPES and L-glutamine, without phenol red) supplemented with 10% (v/v) fetal bovine serum (FBS) and 1x Antibiotic-Antimycotic (Gibco/Life Technologies, Mulgrave, VIC, Australia). Cells were cultured at 37°C in a humidified atmosphere of 5% CO_2_ in air. At approximately 80% confluence cells were detached with trypsin/EDTA Solution (Gibco), washed, counted, and transferred into six-well plates at a density of 0.8 × 10^6^ cells/well. Cultures reaching 50% confluence were incubated with fresh medium with or without 8-Br-cAMP (8-bromoadenosine-3′,5′-cyclic monophosphate, 2.5 × 10^−4^ mol L^−1^) and/or 5-AZA-dC (5-aza-2′-deoxycytidine, 5 × 10^−6 ^mol L^−1^) (Sigma-Aldrich, Sydney, NSW, Australia) followed by harvesting for RNA and DNA isolation. Drug concentrations were optimised in previous studies and showed no toxic effects in BeWo cells at the concentrations and exposure times employed [19, 22, 23]. Each treatment was repeated three times in independent experiments.

### 2.2. RNA Extraction and cDNA Synthesis

RNA was extracted from cells using the RNeasy Mini Kit (Qiagen, Chadstone Centre, VIC, Australia) according to the manufacturer's protocol. RNA was eluted from the RNeasy Spin Columns with 30 *μ*L of RNase-free water and quantified using a NanoDrop 1000 spectrophotometer (Thermo Fisher Scientific Australia, Scoresby, VIC, Australia). Contaminating DNA was removed by DNAse treatment using the TURBO DNA-free kit (Ambion/Life Technologies) following the “Routine” protocol. The total reaction volume was 20 *μ*L including 2 *μ*L of 10x DNase buffer, 1 *μ*L of DNase, and up to 2 *μ*g of RNA. The purified RNA was quantified by UV absorption (NanoDrop 1000). RNA integrity in all samples was assessed by agarose gel electrophoresis.

Prior to reverse transcription, the RNA samples were spiked with 5 × 10^6^ copies of Alien RNA Transcript (supplied with the Alien QRT-PCR Inhibitor Alert kit, Stratagene/Integrated Sciences, Chatswood, NSW, Australia) per microgram RNA. The Alien RNA Transcript served as a reference RNA of equal abundance in all samples and PCR runs [24]. RNA was reverse transcribed using the SuperScript III First-Strand Synthesis System for RT-PCR (Invitrogen) with random hexamer primers.

### 2.3. Real-Time PCR

Real-time PCR was performed using an Applied Biosystems 7500 Real-Time PCR System with reagents supplied by the manufacturer (Applied Biosystems/Life Technologies). The amplification reaction contained template cDNA from 20 ng of reverse-transcribed RNA, 6 × 10^−7^ moles L^−1^ forward and 3 × 10^−7^ moles L^−1^ reverse primer, SYBR Green Master Mix, and MilliQ water to a total volume of 25 *μ*L. The* CRH* cDNA primers were designed and optimised by Sehringer et al. [25] and are listed in Table 1. Primer sequences for amplifying Alien cDNA are proprietary and were used according to the manufacturer's instructions (Stratagen/Integrated Sciences). Amplifications were performed in triplicate. The temperature sequence was 50°C for 2 min, 95°C for 10 min, 40 cycles of 95°C for 15 s, and 60°C for 1 min, followed by melting curve analysis. No-template control and no-reverse transcriptase controls for all samples were included to detect residual genomic DNA. Expression levels of the* CRH* mRNA were determined relative to Alien RNA according to the ΔΔCt method [26].

### 2.4. Genomic DNA Extraction

Genomic DNA was extracted using the QIAamp DNA Mini Kit (Qiagen). Cells grown in six-well plates were collected in 750 *μ*L PBS (phosphate-buffered saline; 137 × 10^−3 ^mol L^−1^ NaCl, 2.7 × 10^−3^ mol L^−1^ KCl, 8 × 10^−3 ^mol L^−1^ Na_2_HPO_4_, and 2 × 10^−3^ mol L^−1^ KH_2_PO_4_, pH 7.4) using cell scrapers and centrifuged for 5 min at 300 ×g. Cell pellets were resuspended in 200 *μ*L of PBS and processed for DNA isolation as per the manufacturer's instructions. Genomic DNA was eluted from the mini spin columns with 200 *μ*L MilliQ water, quantified using the NanoDrop 1000 spectrophotometer, and stored at 4°C.

### 2.5. Bisulfite Treatment, Amplification, and Isolation of the Bisulfite-Converted* CRH* Proximal Promoter Fragment

Up to 300 ng of genomic DNA was bisulfite-converted and purified using the methylSEQr Bisulfite Conversion Kit (Applied Biosystems). The* CRH* proximal promoter regions were PCR-amplified using the TOPTaq Master Mix kit (Qiagen) and two sets of nested primers designed with the Methyl Primer Express Software v.1.0 (Applied Biosystems). The primer sequences are listed in Table 1. PCR reactions contained purified bisulfite-converted DNA template, 25 *μ*L of 2x TOPTaq Master Mix, 4 × 10^−7^ mol L^−1^ of each primer, and MilliQ water up to 50 *μ*L final volume. The conditions for the first PCR amplification included an initial step at 94°C for 3 min, followed by 30 cycles of 94°C for 30 s, 54°C for 30 s, and 72°C for 1 min and a final extension step at 72°C for 10 min. One microliter of a 20-fold diluted aliquot of the first PCR reaction was used as template for the second PCR amplification using the nested primer set. PCR conditions were 94°C 30 min, 30 cycles of 94°C for 30 s, 50°C for 30 s, and 65°C for 1 min and an extension step at 65°C for 10 min.

Following amplification, 20 *μ*L of the PCR reaction mixture was separated by agarose gel electrophoresis and the amplification product was visualised with ethidium bromide. The gel slice containing the amplified DNA fragment was excised, extracted, and purified using the Wizard SV Gel and PCR Clean-Up System (Promega, Auburn, VIC, Australia). The DNA was collected in 50 *μ*L MilliQ water by the centrifugation of the SV Minicolumn. The purified PCR product was quantified by UV absorbance and used for ligation immediately.

### 2.6. Cloning and Sequencing

The bisulfite-converted and PCR-amplified DNA was ligated into the pGEM-T Easy Vector using reagents provided by the manufacturer (Promega). The 10 *μ*L reaction mixtures contained Ligation Buffer, 3 Weiss units of T4 DNA Ligase, 50 ng of pGEM-T Easy Vector, and PCR product at 3 : 1 insert: vector molar ratio. A positive control using the control DNA provided and a negative control (no PCR product) were also included. The reaction mixtures were incubated at 4°C overnight. The ligation mixture was used subsequently to transform JM109 Competent Cells (Promega) according to the manufacturer's protocol. Fifty *μ*L of transformed cell suspension was spread onto duplicate LB/ampicillin/IPTG/X-Gal plates and incubated at 37°C overnight. At least 20 white colonies were randomly picked and streaked individually on new plates. The white streak colonies were picked the next day and cultured in 5 mL Luria Broth at 37°C overnight. Plasmids were isolated from the minicultures using the GenElute plasmid Miniprep Kit (Sigma-Aldrich, Castle Hill, NSW, Australia). Plasmid DNA purity and yield were assessed by UV absorption.

The presence of inserts was verified by digesting an aliquot from each plasmid preparation with EcoR I (Promega) followed by agarose gel electrophoresis. Plasmids containing the expected size inserts (≈300 bp) were sequenced from both directions by the Australian Genome Research Facility (AGRF, Brisbane, QLD, Australia). The sequencing primers were designed by Promega and produced by Invitrogen/Life Technologies (forward: 5′-TATTTAGGTGACACTATAG-3′, reverse: 5′-TATTTAGGTGACACTATAG-3′). Methylation patterns were determined using the BiQ Analyzer software [27]. Quality control was automatically performed and any sequence with an unacceptably low conversion rate or high number of sequencing errors was excluded. The program also generated lollipop-style diagrams of the methylation patterns.

### 2.7. Statistical Analysis

*CRH* mRNA relative abundance values were logarithmically transformed to approach normal distribution. Group comparisons were performed by *t*-tests. Ten randomly selected clones, representing individual gene copies, were processed for methylation frequency analyses from each DNA sample using the Chi-square test and Fisher's exact test as appropriate. Significance was determined at *p* < 0.05. If not specified, two-sided test results are shown. Significant one-sided tests are reported in cases when the two-sided tests showed borderline significance. The STATA (College Station, TX, USA) software package was used for the statistical calculations.

## 3. Results

### 3.1. Time Course of* CRH* mRNA Expression

**Figure 1:**
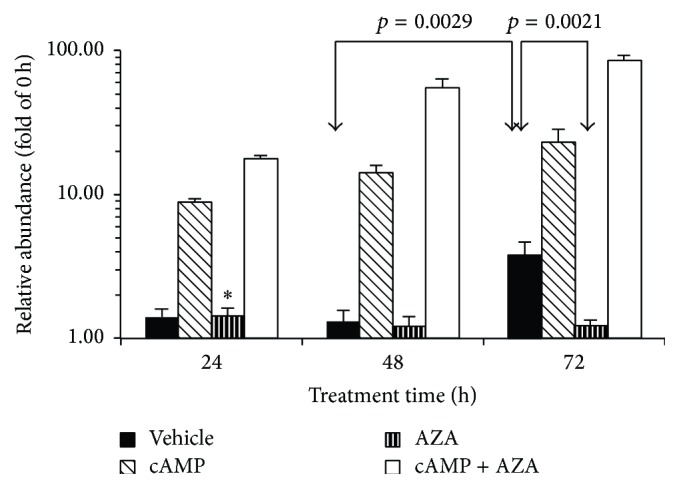
*CRH* mRNA relative abundance in BeWo cells treated with 8-Br-cAMP (cAMP, 2.5 × 10^−4^ mol L^−1^) and 5-AZA-dC (AZA, 5 × 10^−6^ mol L^−1^) for 24 h, 48 h, and 72 h. The average ± SEM of results of four independent experiments is shown. Significant pairwise differences (*t*-test) between vehicle-treated and 5-AZA-dC-treated cultures are indicated. Significant pairwise differences involving 8-Br-cAMP-treated cultures and 8-Br-cAMP + 5-AZA-dC-treated cultures are described in the Results. ^∗^Significantly higher than 0 h (*p* = 0.0276, one-sample, one-sided *t*-test).

*CRH* mRNA was detectable in the BeWo cells. As shown in Figure 1, abundance was not significantly different between 0 h and 24 h and between 24 h and 28 h of incubation. Between 48 h and 72 h, a 2.9-fold increase (*p* = 0.029) was observed, which was coincident with the reported spontaneous syncytialisation of BeWo cells [19]. In the presence of 8-Br-cAMP (2.5 × 10^−4^ mol L^−1^), a powerful stimulant of syncytial differentiation [19], significant increases of* CRH* mRNA level were detected between 0 h and 24 h (*p* < 0.0001), 24 h and 48 h (*p* = 0.03), and 48 h and 72 h (*p* = 0.049, one-sided *t*-test) with a maximum at 72 h, which was 23.2-fold higher than the 0 h level (Figure 1). In cultures treated with the DNA methyltransferase inhibitor 5-AZA-dC (5 × 10^−6 ^mol L^−1^) a slight, but significant, increase of* CRH* mRNA abundance was observed between 0 h and 24 h (1.43-fold, *p* = 0.028, one-sided *t*-test), but there was no further change between 24 h and 48 h and between 48 h and 72 h. Combined treatment with 8-Br-cAMP and 5-AZA-dC resulted in robust increases of* CRH* mRNA levels between 0 h and 24 h (*p* < 0.0001), 24 h and 48 h (*p* = 0.0026), and 48 h and 72 h (*p* = 0.039) reaching a 86.4-fold rise at 72 h compared to 0 h (Figure 1).

### 3.2. Effects of 8-Br-cAMP and 5-AZA-dC on* CRH* mRNA Expression

8-Br-cAMP increased* CRH* mRNA abundance relative to vehicle at all time points (24 h, *p* = 0.0042; 48 h, *p* = 0.0066; 72 h, *p* = 0.0004; Figure 1), which confirmed previous findings of the stimulatory effects of 8-Br-cAMP on* CRH* gene expression in BeWo cells [28]. 5-AZA-dC treatment had no effect at 24 h and 48 h and reduced* CRH* mRNA abundance relative to vehicle at 72 h, effectively blocking the increase seen between 48 h and 72 h in vehicle-treated cells (*p* = 0.0021, Figure 1). Combined treatment with the cyclic nucleotide and the DNA methyltransferase inhibitor upregulated* CRH* mRNA expression at all time points beyond the level reached in response to 8-Br-cAMP alone (24 h, *p* < 0.0001; 48 h, *p* = 0.0002; 72 h, *p* = 0.0029; Figure 1).

### 3.3. Methylation of the* CRH* Promoter

**Figure 2:**
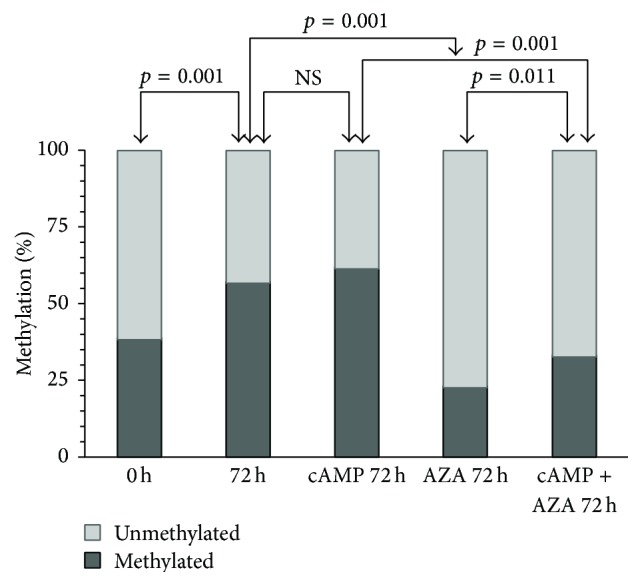
Methylation of the* CRH* promoter. Percent methylation of the 9 CpG sites together was calculated by combining three independent experiments and ten randomly selected clones (individual gene copies) sequenced per treatment in each experiment. Methylation frequencies were compared by Fisher's exact test. Statistical comparisons (*p* < 0.05, significant and NS, not significant) are shown by the arrows. 0 h, no treatment; 72 h, 72 h of culture; cAMP 72 h, treatment with 2.5 × 10^−4^ mol L^−1^ of 8-Br-cAMP for 72 h; AZA 72 h, treatment with 5 × 10^−6 ^mol L^−1^ 5-AZA-dC for 72 h; cAMP + AZA 72 h, combined treatment with 8-Br-cAMP and 5-AZA-dC for 72 h.

The significant effects of 5-AZA-dC on* CRH* mRNA expression suggested that DNA methylation was involved in the control of* CRH* gene activity. To explore this further, we have determined the effects of 8-Br-cAMP and 5-AZA-dC on the methylation of the 9 CpG sites present in the* CRH* proximal promoter. Bisulfite sequencing revealed partial methylation (38% of the 9 CpG sites combined) at 0 h, before treatments commenced (Figure 2), which increased spontaneously to 57% (*p* = 0.001) by 72 h of culture. Treatment with 8-Br-cAMP (250 *μ*M) resulted in a similar increase of methylation (to 61%), not significantly different from the vehicle-treated control. Treatment with 5-AZA-dC for 72 h reduced promoter methylation to 23%, which was significantly less than the control (*p* = 0.001). Combined treatment with 5-AZA-dC and 8-Br-cAMP increased the level of methylation compared to 5-AZA-dC alone (to 33%, *p* = 0.011) but did not reach the methylation level observed in cells treated with 8-Br-cAMP only (*p* = 0.001, Figure 2).

### 3.4. Methylation of the Individual CpG Sites in the* CRH* Proximal Promoter

**Figure 3:**
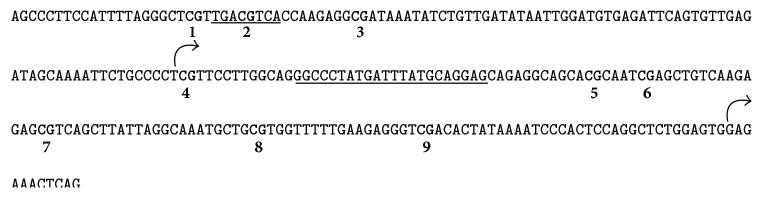
DNA sequence of the* CRH* proximal promoter. The methylatable CpG dinucleotides are in boldface and are numbered 1 to 9. The canonical CRE, which contains CpG2, and the noncanonical cAMP-response regions are underlined. The two major transcription initiation sites are flagged by the arrows.

**Figure 4:**
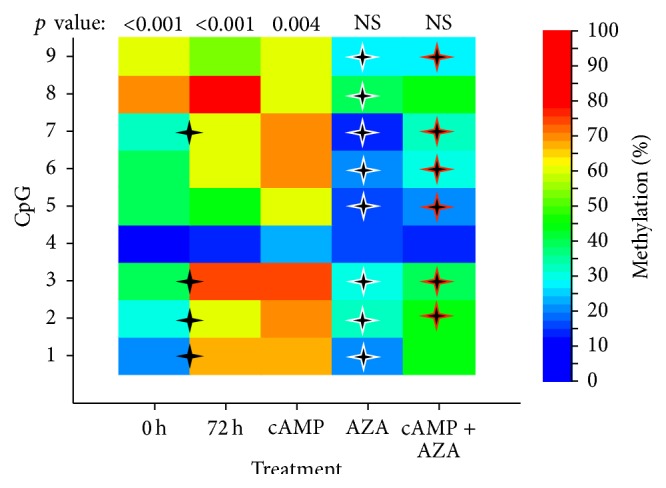
Methylation of individual CpG dinucleotides at 0 h and 72 h of culture (0 h and 72 h, resp.), after 72 h with 8-Br-cAMP (cAMP) and after 72 h with 5-AZA-dC (AZA), and the combination of the two (cAMP + AZA). Treatments are denoted on the horizontal axis and CpG numbers (shown in Figure 3) are indicated on the vertical axis. The heatmap shows % methylation according to the scale on the right. Three independent experiments with ten clones (randomly selected individual gene copies) sequenced at each time point were combined to calculate % methylation. The statistical significance of methylation frequency differences among the CpG sites at each treatment condition is shown on the top (Fisher's exact test). The black crosses indicate significantly different methylation of CpGs 1, 2, 3, and 7 between 0 h and 72 h; crosses with white lining in the AZA fields denote significant CpG-site-specific differences compared to 72 h, and crosses with red lining in the cAMP + AZA fields denote significant differences of CpG site methylations as opposed to cAMP treatment alone (*p* < 0.05, Fisher's exact test).

**Figure 5:**
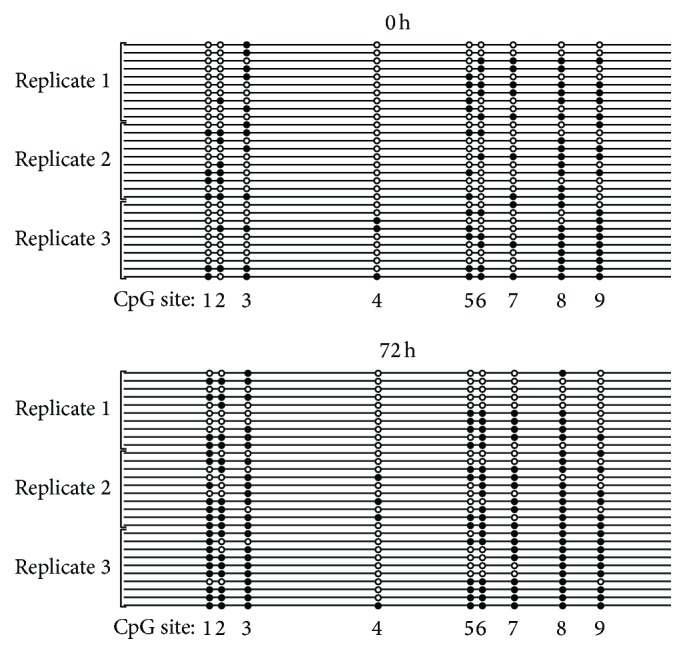
CpG methylation in individual copies of the* CRH* proximal promoter. Three replicate sets of BeWo cells were processed for clonal bisulfite sequencing at the 0 h (upper panel) and 72 h (lower panel) treatment times. Ten randomly selected clones were sequenced from each culture. Each line represents one copy of the promoter with open and closed circles denoting unmethylated and methylated CpGs, respectively. CpG site numbers as shown in Figure 3 are also indicated.

Clonal bisulfite sequencing determines cytosine methylation with single base resolution in individual alleles (gene copies). The technique enabled us to determine the particular CpG sites that undergo methylation changes under the treatment conditions that influence methylation levels overall, as presented in Figure 2. The scheme in Figure 3 shows the positions of the 9 methylatable CpG dinucleotides in the human* CRH* proximal promoter. The two major transcription initiation sites and the two sequence regions implicated in the cAMP-response are also indicated with CpG2 residing within the CRE [8–11, 29]. The heatmap in Figure 4 illustrates the methylation levels of the 9 CpGs under the treatment conditions employed. Methylation levels were significantly different among the CpG sites ranging from 10% to 70% at 0 h and from 13% to 80% at 72 h of culture (*p* < 0.001, Figure 4) indicating site-specific differential methylation. The methylation level of CpGs 1, 2, 3, and 7, but not of CpGs 4, 5, 6, 8, and 9 increased significantly during the 72 h culture period demonstrating that methylation was dynamic at these sites.  Figure 5 shows the methylation of each CpG in the cloned copies of the* CRH* proximal promoter. The scattered distribution of methylated and unmethylated CpG sites suggests that the partial methylation observed was allele independent both at 0 h and at 72 h of culture. Cells treated with 8-Br-cAMP and 5-AZA-dC had similar scattered distribution of methylated CpGs in individual alleles (not shown).

In cells treated with 8-Br-cAMP for 72 h, the CpG sites remained differentially methylated (from 23% to 73%, *p* = 0.004), and the methylation levels of individual CpGs were not significantly different from the corresponding sites in the vehicle-treated control (Figure 4). Treatment with the DNA methyltransferase inhibitor 5-AZA-dC decreased methylation at all CpG sites compared to vehicle, except for CpG 4, where methylation was relatively low. Furthermore, 5-AZA-dC abolished the differences between the methylation levels of the individual CpGs. The methyltransferase inhibitor eliminated the methylation differences among individual CpGs in the presence of 8-Br-cAMP as well (Figure 4). Cotreatment with 8-Br-cAMP prevented, however, the demethylating action of 5-AZA-dC at CpGs 1, and 8, but not at CpGs 2, 3, 5, 7, and 9 (cAMP* versus* cAMP + AZA in Figure 4). Finally, no individual CpG site exhibited a statistically significant methylation difference in 8-Br-cAMP + 5-AZA-dC-treated cells compared to treatment with the methyltransferase inhibitor alone (AZA* versus* cAMP + AZA in Figure 4) despite the small, but significant, overall increase in methylation (AZA 72 h* versus* cAMP + AZA 72 h, in Figure 2).

## 4. Discussion

The aim of this study was to explore the involvement of promoter methylation in* CRH* gene regulation in human trophoblast cells. Placental* CRH* expression is predictive of gestational length and is influenced by pregnancy disorders [4, 30]. DNA methylation is a developmentally regulated epigenetic modification influenced by environmental inputs [31, 32], which can potentially control* CRH* gene expression during pregnancy and in response to pathogenic factors. In our experiments we used the choriocarcinoma-derived BeWo cell line, which is a well-characterized trophoblast model exhibiting increased* CRH* gene expression during spontaneous and cAMP-induced syncytial differentiation similar to normal trophoblasts [19]. Our DNA sequencing data show that the* CRH* proximal promoter sequence is identical in BeWo cells and in normal trophoblasts including all reported transcription factor response elements and the methylation sensitive CpG sites (Figure 3). We have shown by clonal bisulfite sequencing that the CpGs within the* CRH* proximal promoter are partially methylated with significant differences among the individual sites. The size of the analysed sequence (261 bp) and the methylation level correspond to an “intermediate methylation region,” which is a genomic feature implicated in tissue specific epigenetic gene regulation [33]. This is similar to normal trophoblasts, which also exhibit allele independent partial and differential methylation in the* CRH* promoter region [34]. DNA methylation increases in BeWo cells during culture and the DNA methyltransferase inhibitor 5-AZA-dC changes cell phenotype and gene expression levels [21, 22, 35]. Our results show that the* CRH* promoter follows this general trend, which is different from primary trophoblasts, where* CRH* promoter methylation does not change in culture and remains unaltered by 5-AZA-dC and 8-Br-cAMP under the conditions where BeWo cells show methylation changes [34]. For this reason, BeWo cells are uniquely suited to explore the relationship between* CRH* promoter methylation level and gene activity.

Methylation of CpG island promoters is associated generally with the repression of gene activity [12, 14, 21]. This also applies to “CpG island shore” regions, which exhibit tissue specific methylation inversely related to gene expression [18]. The* CRH* promoter is located in a CpG island shore region relative to a* CRH* intragenic CpG island (chr8:67,089,250-67,089,962 in the GRCh37/hg19 assembly, UCSC genome browser). Our results showed, however, that neither the spontaneous nor the 8-Br-cAMP-evoked increase of* CRH* gene activity was associated with the demethylation of the* CRH* promoter (Figures 1 and 2). Treatment with 5-AZA-dC decreased* CRH* promoter methylation and abolished the CpG site-specific methylation differences as expected (Figures 2 and 4), but it also blocked the spontaneous increase of* CRH* gene expression in culture (Figure 1). 8-Br-cAMP-stimulated* CRH* expression strongly in the presence of 5-AZA-dC (Figure 1), but the cyclic nucleotide actually increased methylation in 5-AZA-dC-treated cells (AZA 72 h* versus* cAMP + AZA 72 h in Figure 2). Thus,* CRH* expression was directly, and not reciprocally, related to promoter methylation under these conditions. This relationship may be unexpected in view of the well-documented global association between gene repression and promoter methylation, but genome wide trends do not necessarily predict the behavior of individual genes. In fact, the methylation level of the CpG-poor class of promoters was found to be uncorrelated with gene activity [36]. The* CRH* promoter falls into the CpG-poor class according to established criteria [36], with partial methylation in both the hypothalamus and the trophoblast [34, 37, 38] (Figure 2). Methylation reduces* CRH* gene expression in the hypothalamus as expected; in trophoblastic BeWo cells, however, promoter methylation appears to have the opposite effect as detailed before. There is evidence to suggest that this cell-specific regulation may result from the methylation-dependent change of the functional properties of the cAMP-response element (CRE) in the* CRH* promoter (Figure 3) [16]. The CRE is critical in regulating the activity of transfected* CRH* promoter-reporter constructs in trophoblast cells [9–11]. It contains a CpG dinucleotide that, when methylated, reduces the affinity of CRE to its cognate transcription factor, CREB, and renders it unresponsive to cAMP-stimulation [15, 39]. At the same time, the methylated CRE has increased affinity to bind the transcription factor C/EBP-alpha, which often activates tissue specific genes during differentiation [16]. The CpG within the CRE was 30% methylated under basal (0 h incubation) conditions (CpG2 in Figure 4). Culturing for 72 h increased CpG2 methylation to 60% concomitantly with enhanced gene expression, while treatment with 5-AZA-dC reduced CpG2 methylation to 23% and diminished* CRH* gene activity (Figures 1 and 4). Considering that C/EBP-alpha is expressed in BeWo cells [40], it is reasonable to conjecture that methylation-evoked changes in the transcription factor binding specificity of the CRE may have contributed to the enhanced* CRH* expression observed in association with increased promoter methylation.

The CpG2 in the CRE was partially methylated under all experimental conditions, which suggests that the unmethylated portion could have mediated stimulation by 8-Br-cAMP using the canonical, CREB-dependent pathway. Moreover, the* CRH* proximal promoter contains a second, noncanonical cAMP-response element (Figure 3), which contributes to the regulation of the gene specifically in the trophoblast [9, 11]. This regulatory sequence does not contain a CpG dinucleotide and does not bind CREB [11]. Methylation of the CRE may thus function to influence the relative contribution of the two cAMP-response elements, their associated transcription factors, and the coupled signaling pathways to the overall activity of the* CRH* gene under basal and cAMP-stimulated conditions.

Cotreatment with 5-AZA-dC strongly augmented the stimulation of* CRH* mRNA expression by 8-Br-cAMP, although the DNA methyltransferase inhibitor alone had no stimulatory effect under the same culture conditions (Figure 1). Cotreatment with 5-AZA-dC, however, decreased CpG methylation in the CRE (CpG2, Figure 4) to 43.3% from 70% measured after 8-Br-cAMP treatment (*p* = 0.034, Fisher's exact test, one-sided). The increased proportion of unmethylated CREs in the cell population could explain, at least partially, the augmented response to 8-Br-cAMP-stimulation. Moreover, 5-AZA-dC decreases DNA methylation globally increasing or repressing the activity of numerous genes [41–43]. This suggests the possibility that 5-AZA-dC may potentially influence* CRH* expression indirectly through intervening gene products generating synergy between 8-Br-cAMP and 5-AZA-dC. Although gene activation occurs in 5-AZA-dC-treated BeWo cells [22, 23, 35], it remains to be established whether transcription factors or other gene products controlled by DNA methylation regulate* CRH* expression in trophoblasts.

## 5. Conclusions

We have utilized the dynamic changes of DNA methylation in the BeWo cell line to explore the relationship between this epigenetic chromatin modification and* CRH* gene expression in a trophoblastic cell type. Clonal bisulfite sequencing revealed the CpG site-specific and allele independent partial methylation of the* CRH* proximal promoter and classified it as an intermediately methylated region of the genome. The data suggest that promoter methylation determines the contribution of the CRE, its various associated transcription factors, and a trophoblast specific alternative cAMP-response element to* CRH* gene regulation. Furthermore, our results are consistent with the possibility that DNA methylation controls* CRH* expression indirectly, but any intervening factor that may regulate* CRH* expression by a DNA methylation-dependent mechanism remains to be determined.

## Figures and Tables

**Table 1:** Primers used for quantitative real-time RT-PCR (qRT-PCR) and bisulfite sequencing.

Primers for CRH mRNA qRT-PCR		
Forward	5′-TCCCATCTCCCTGGATCTCAC-3′	GeneBank number NM_00756
Reverse	5′-GTGAGCTTGCTGTGCTAACTGCT-3′	

Primers for CRH promoter bisulfite sequencing		

1st PCR	Forward	5′-TTTGGGAAATTTTATTTAAGAATTTTT-3′
	Reverse	5′-CTAAATTTCTCCACTCCAAAACCTA-3′
2nd (nested) PCR	Forward	5′-GTTAATGGATAAGTTATAAGAAGTTTTT-3′
	Reverse	5′-TCCACTCCAAAACCTAAAATAAAAT-3′
